# Genetic dissection of growth trajectories in forest trees: From FunMap to FunGraph

**DOI:** 10.48130/FR-2021-0019

**Published:** 2021-11-03

**Authors:** Li Feng, Peng Jiang, Caifeng Li, Jinshuai Zhao, Ang Dong, Dengcheng Yang, Rongling Wu

**Affiliations:** 1 Center for Computational Biology, College of Biological Sciences and Technology, Beijing Forestry University, Beijing 100083, China; 2 Center for Statistical Genetics, Departments of Public Health Sciences and Statistics, The Pennsylvania State University, Hershey, PA 17033, USA

**Keywords:** Growth, Growth equation, Forest tree, Genetic architecture, Functional mapping, Functional graphing

## Abstract

Growth is the developmental process involving important genetic components. Functional mapping (FunMap) has been used as an approach to map quantitative trait loci (QTLs) governing growth trajectories by incorporating growth equations. FunMap is based on reductionism thinking, with a power to identify a small set of significant QTLs from the whole pool of genome-wide markers. Yet, increasing evidence shows that a complex trait is controlled by all genes the organism may possibly carry. Here, we describe and demonstrate a different mapping approach that encapsulates all markers into genetic interaction networks. This approach, symbolized as FunGraph, combines functional mapping, evolutionary game theory, and prey-predator theory into mathematical graphs, allowing the observed genetic effect of a locus to be decomposed into its independent component (resulting from this locus’ intrinsic capacity) and dependent component (due to extrinsic regulation by other loci). Using FunGraph, we can visualize and trace the roadmap of how each locus interact with every other locus to impact growth. In a population-based association study of Euphrates poplar, we use FunGraph to identify the previously neglected genetic interaction effects that contribute to the genetic architecture of juvenile stem growth. FunGraph could open up a novel gateway to comprehend the global genetic control mechanisms of complex traits.

## Introduction

Because of its fundamental importance in understanding biological processes, growth, i.e., change in the size of an organism with age, has intrigued biologists for centuries^[1−4]^. Growth traits are particularly important to tree breeding because stemwood production heavily depends on how much a tree can grow at a harvest age and how fast a tree grows to reach the targeted amount of harvested wood^[5,6]^. The past decades have witnessed the widespread application of molecular and biotechnological approaches to mapping specific genes, known as quantitative trait loci (QTLs), responsible for phenotypic variation in stemwood growth^[7−13]^. Growth traits can be better mapped by FunMap, a statistical method that integrates the time dimension of trait development into a genetic mapping or association setting, remarkably improving the statistical power and biological interpretations of QTL detection^[14−20]^.

Like all existing approaches, FunMap tests and characterizes individual key QTLs based on the marginal effects of individual markers. Extensive analysis of genome-wide association study (GWAS) data suggests that complex traits are simultaneously controlled by all genes the organism may carry throughout its entire genome^[21]^. It becomes increasingly clear that complex traits may not only be determined by the independent actions of individual genes, but also by their epistatic interactions through an intricate but well-organized network^[21−25]^. Unfortunately, current theory and methods have limited power to disentangle the complexity of epistasis^[26]^. First, traditional approaches detect epistasis based on an exhaustive search of marginal pairwise interactions^[15,27]^, failing to portray a systematic characterization of all possible, simultaneously occurring epistasis. Second, widely used mapping or association studies can estimate the magnitude and sign of epistasis, but cannot identify its direction by which one gene regulates the other. Third, the requirement of sample size to chart a systematic picture of interactomes containing a vast number of gene-gene combinations cannot be met in practice. For example, under reasonable assumptions in human association studies, the detection of significant epistasis between a single pair of genes would need as many as 50,000 samples^[28]^.

More recently, our group has developed a computational model for reconstructing a multilayer, omnigenic interactome network of SNPs from a high-density linkage map or GWAS^[29−33]^. There is a rich body of literature on reconstructing gene regulatory networks from expression data^[34−37]^, but there is little methodology to infer genetic networks from genotype and phenotype data. Our model fills this gap through integrating functional mapping and evolutionary game theory and prey-predator theory and taking advantage of time dimension to augment information for interaction modeling. By viewing genetic architecture as an evolving game, we introduce the notion of evolutionarily stable strategy^[38]^ to derive a system of nonlinear Lotka-Volterra prey-predator equations. Through these equations, we decompose the marginal effect of each locus estimated from functional mapping into its two underlying components: the *independent* effect that occurs when this locus is assumed to be in isolation and the *dependent* effect due to the joint influence of other loci. We code independent effects of individual loci as nodes and dependent effects of individual locus pairs as edges into graphs. These graphs, i.e., FunGraph, are filled with bidirectional, signed, and weighted epistasis^[39]^ and allowed to change with time, thus providing a means of addressing many fundamental questions, such as how each locus affects growth on its merit, how a locus regulates, or is regulated by, other loci, and how loci change their genetic effects over spatiotemporal scales.

In this article, we describe our FunGraph and disseminate it into a broader community of forestry. FunGraph is characterized by several key steps, including functional mapping, evolutionary game theory-based prey-predator modeling, functional clustering, and network reconstruction. We review each step and show a smooth transition from one step to the next. We analyze a GWAS data from the salt-resistant experiment of Euphrates poplar collected from its natural distribution. We conclude by discussing the advantages and disadvantages of FunGraph, providing the recommendations of its use in practical mapping or association studies.

## Model Review

### Functional mapping

Many mapping approaches perform associations between genotype and phenotype measured at single time points. Different from this static mapping strategy, FunMap incorporates growth equation into a mapping framework, allowing the temporal pattern of genetic effects to be characterized^[14,16]^. FunMap has been widely used to map growth QTLs in a variety of species^[15,17-20]^. To help readers of this journal better understand FunMap, we outline several key steps for its derivations. Consider a tree mapping population composed of *n* individuals which have been genotyped through the entire genome to produce a high-dimensional SNP data. In this population, let $ {\mathbf{t}}_{i}=({t}_{i1},\dots ,{t}_{i{T}_{i}}) $ denote the measure schedule of a growth trait for individual *i* (*i* = 1, …, *n*). Let $ {\mathbf{y}}_{i}=\left({y}_{i}\left({t}_{i1}\right),\dots ,{y}_{i}\left({t}_{i{T}_{i}}\right)\right) $ denote the phenotypic values of individual *i* measured per to a time schedule. Assume that there are *J* genotypes at a locus with observations denoted as *n*_1_, …, *n*_*J*_, respectively. The likelihood of trait values at a given molecular marker is expressed as

1 $ L\left(y\right)=\prod \limits_{j \,=\, 1}^{J}\prod\limits _{i \,=\, 1}^{{n}_{j}}{f}_{j}\left({\mathbf{y}}_{i}\right)$

where $ {f}_{j}\left({\mathbf{y}}_{i}\right) $ is the multivariate normal distribution of individuals, with mean vector for individual *i* carrying genotype *j* expressed as

2 $ {\mathbf{\mu }}_{ji}=\left({\mu }_{j}\left({t}_{i1}\right),\dots ,{\mu }_{j}\left({t}_{i{T}_{i}}\right)\right) $

and individual-specific covariance matrix **Σ**_*I*_, which is common to different genotypes.

FunMap models the structures of the mean vector (2) and covariance matrix^[14,16]^. For a trait growing during a period of development, we often use a logistic equation to describe the form of growth. For marker genotype *j* on individual *i*, its age-varying mean value (at age *τ*) is modeled by

3 $ {\mu }_{j}\left({t}_{i\tau }\right)=\frac{{a}_{j}}{1+{b}_{j}{e}^{-{r}_{j}{t}_{i\tau }}} $

where growth parameters (*a*_*j*_, *b*_*j*_, *r*_*j*_) are the asymptotic growth, initial growth, and relative growth rate of genotype *j*. By estimating and testing these parameters, we can infer how a genetic marker is associated with growth trajectory.

Because covariance matrix ${\Sigma}_{i}$ obeys some autocorrelative structure, several time-series models have been used to model the covariance structure, including the first-order autoregressive model (AR(1))^[14]^ and first-order structured antedependence model (SAD(1))^[40]^. The AR(1) model produces analytical solutions for the determinant and inverse of the covariance matrix, which can increase computational efficiency, but it needs stationarity assumptions difficult to meet in a real situation. Zimmerman and Núñez-Antón^[41]^ derived the closed forms of solving the determinant and inverse of the covariance matrix for the SAD(1) model that does not rely on the stationarity assumptions. As a result, we incorporated the SAD(1) model to structure ${\Sigma}_{i}$ by using two parameters, antedependent parameter (describing how much the residual at one age depends on that at the preceding age) and innovative variance (i.e., the variance produced at a specific age).

The maximum likelihood estimates of genotype-dependent growth parameters and covariance-structuring parameters can be obtained by a simplex algorithm^[42]^. Whether a genetic marker is associated with growth trajectory can be tested on the basis of the following hypotheses

4 \begin{equation*}\begin{split}&
{{\mathrm{H}}_0}  :{\mathrm{ }}\left( {{a_j},{b_j},{r_j}} \right) \equiv \left( {a,b,r} \right)\\
&{{\mathrm{H}}_1}  :{\mathrm{ At}}\;\;{\mathrm{ least }}\;{\mathrm{one }}\;{\mathrm{of }}\;{\mathrm{the}}\;{\mathrm{ equalities}}\;{\mathrm{ in}}\;{\mathrm{ the}}\;{\mathrm{ }}{{\mathrm{H}}_{\mathrm{0}}}\;{\mathrm{does}}\;{\mathrm{ not }}\;{\mathrm{hold}}
\end{split}\end{equation*}

where the test statistic is calculated as the log-likelihood ratio (LR) of the full model (H_1_) over the reduced model (H_0_). The LR may be regarded as being chi-square distributed. The genome-wide significance level of a variant is corrected for multiple comparisons. Empirically, the critical threshold for claiming the genome-wide existence of a significant marker can also be determined from permutation tests.

For a given locus *s* (*s* = 1, …, *m*) with *J* genotypes, we estimate its age-varying genetic standard deviation (at age $ \tau $) as a proxy to the genetic effect of this locus using the following equation

5 $
 {z}_{s}\left(\tau \right)=\sqrt{\frac{1}{n}\sum \limits_{j\,=\,1}^{J}{n}_{j}{\left[{\mu }_{j}\left(\tau \right)-{\stackrel-{\mu }}_{s}\left(\tau \right)\right]}^{2}}
$

where $ {\mu }_{j}\left(\tau \right) $ is calculated from the growth equation (3) using the estimated growth parameters (*a*_*j*_, *b*_*j*_, *r*_*j*_), and $ {\stackrel{-}{\mu }}_{s}\left(\tau \right)=  \displaystyle\sum _{j\,=\,1}^{J}\dfrac{{n}_{j}}{n}{\mu }_{j}\left(\tau \right) $ is the overall mean of trait values calculated on the basis of genotypes at marker *s*. Equation (5) can be used to visualize the developmental pattern of genetic variance for a given trait.

### Nonlinear prey-predator modelling of evolutionary game theory

We view *m* markers as a system in which different genes interact with each other to affect phenotypic traits through the lens of evolutionary game theory. We argue that the genetic variance of a marker is determined not only by its own intrinsic capacity but also by the interactions of other markers with this marker. Based on this consideration, we derive a system of nonlinear Lotka-Volterra-based ordinary differential equations (ODEs), expressed as

6 $ \frac{d{z}_{s}\left(\tau \right)}{dt}={Q}_{s}\left({z}_{s}\left(\tau \right):{\mathrm{\Phi }}_{s}\right)+\sum \limits_{{s}{'}=1,{s}{'}\ne s}^{m}{Q}_{s{s}{'}}\left({z}_{{s}{'}}\left(\tau \right):{\mathrm{\Phi }}_{s{s}{'}}\right),\;\;s=\mathrm{ }1,\mathrm{ }\dots ,m $

where the time-varying change of the (epi)genetic variance of marker *s* is decomposed into two components: the first is called the independent genetic variance derived from this marker’s endogenous action, specified by $ {Q}_{s}(· ) $, and the second is called the dependent genetic variance resulting from the aggregate exogenous regulation of other *d*_*s*_ markers, specified by $\sum {Q}_{s{s}{'}}(· )$. Note that *d*_*s*_ (*d*_*s*_ << *m*) is determined by variable selection. We implement a marker-specific smoothing function to model $ {Q}_{s}(· ) $, and $ {Q}_{s{s}{'}}(· ) $, respectively. By estimating a set of ODE parameters $ {\mathrm{\Phi }}_{s} $, we can determine the pattern and magnitude of the independent (epi) genetic variance of individual markers. Similarly, the estimation of a set of ODE parameters $ {\mathrm{\Phi }}_{s{s}{'}} $ enables us to characterize whether and how the genetic variance of marker *s* depends jointly on other markers ($ s $ = 1, …, *m*; $ {s}{'}=1,\mathrm{ }\dots ,s-1,s+1,\mathrm{ }\dots ,m $). Because of their infinitely differentiable property, we choose Legendre Orthogonal Polynomials (LOP) to fit the smoothing functions^[43]^.

In practice, it is not possible that each marker interacts with every other marker in the whole system, rather one marker may interact with a small subset of markers; i.e., each marker may be regulated by a small set of regulators. To choose such a small set of regulators for each marker, we incorporate a LASSO-based variable selection procedure. LASSO is built on a LOP-based nonparametric regression derived from ODEs of equation (6) by which the genetic effect of a SNP is expressed as the function of its independent effect and dependent effects due to other SNPs. This variable selection allow us to choose a small set of the most significant SNPs (*d*_*s*_ << *m*) that link with a focal SNP *s*. After *d*_*s*_ is determined, we reduce ODEs of equation (6) to include the summation of dependent effects due to only *d*_*s*_ SNPs.

We implement the fourth-order Runge-Kutta algorithm to solve the reduced ODEs by a non-linear least squared approach. After ODE parameters are estimated, we encapsulate independent variances of different markers $ {Q}_{s}(· ) $ as nodes and dependent variances of different marker pairs $ {Q}_{s{s}{'}}(· ) $ as edges into a mathematical graph. Because $ {Q}_{s{s}{'}}(· ) $ has directionality and reciprocity, is quantitative, and can be positive or negative, this graph provides a bidirectional, signed, and weighted interaction network that characterizes a complete picture of genetic architecture determined by all genes under consideration.

### Reconstructing genetic networks

**Figure 1:**
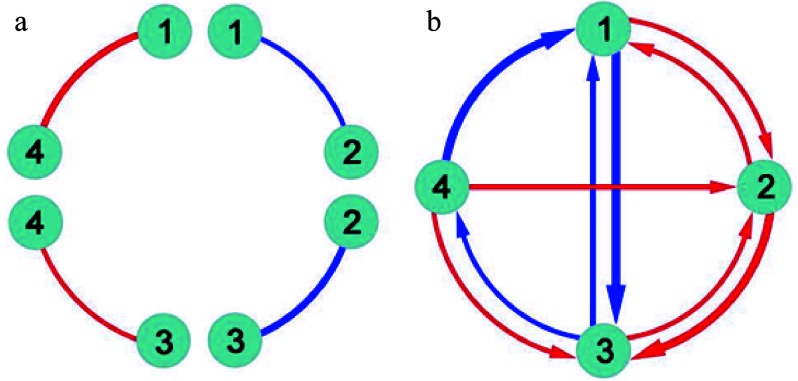
Detection of epistasis by (a) classic quantitative genetic approaches and (b) functional network mapping. Four loci are assumed, with pairwise interactions being shown. Classic approaches can only estimate the sign and strength of epistasis, but fail to identify the direction of epistasis. Red and blue lines denote positive and negative epistasis, respectively, with the thickness proportional to the strength of epistasis.

The functional network mapping model partitions the marginal genetic effect of a marker into its underlying independent and dependent components and codes the independent effects as nodes (main effects) and the dependent effects as edges (epistatic effects) into mathematical networks. Classic quantitative genetic approaches estimate and test pairwise genetic interactions (Fig. 1a), failing to jointly characterize the direction, sign, and weight of epistasis. Functional network mapping can not only fully capture these features of epistasis, but also visualize a systematic network of epistasis among all loci (Fig. 1b). Specifically, functional network mapping classifies epistasis into seven qualitative types as follows:

● *Symmetric positive epistasis*, by which two loci activate each other to the same extent;

● *Asymmetric positive epistasis*, by which two loci activate each other but to different extents;

● *Directional positive epistasis*, by which one locus activates the other but the second has no effect on the first;

● *Symmetric negative epistasis*, by which two loci suppress each other to the same extent;

● *Asymmetric negative epistasis*, by which two loci suppress each other but to different extents;

● *Directional negative epistasis*, by which one locus suppresses the other but the second has no effect on the first;

● *Altruistic/repressive epistasis*, by which one locus activates the other but the second suppresses the first.

Each of these epistatic types can be quantitatively measured (Fig. 1b). Despite their distinct biological relevance, these types are collectively referred to as the overall concept of epistasis.

### Detecting network communities by functional clustering

A genetic mapping study generally involves a huge number of SNPs that lead to an astronomically large number of pairwise links. However, complete interconnections of all SNPs may not exist because this does not help the network buffer against stochastic perturbations^[44]^. For this reason, genetic networks, like many other types of networks, such as ecological networks and social networks, are sparse and patchy, forming distinct network communities. To detect such communities from a large-scale network, Wu and Jiang (2021) implement functional clustering^[45,46]^ to split all SNPs into their distinct modules in each of which SNPs are more similar to each other in terms of their genetic effect temporal pattern than to those from other modules. The optimal number of modules for a given pool of SNPs is determined on the basis of parsimonious information criteria, such as AIC or BIC^[30]^. Thus, SNPs within a module form relatively strong interconnections as distinct network communities. If the number of SNPs within a module is still too large to prevent the reconstruction of their interconnected network, we may cluster it into distinct submodules. Likewise, if a submodule is still too big, we further cluster it into distinct sub-submodules according to an information criterion. This tree-like clustering procedure continues until the number of SNPs within a unit reaches a number that is tractable for SNP-SNP network reconstruction.

The above steps allow us to reconstruct a multilayer, tree-like, sparse, omnigenic interactome network. The term 'multilayer' implies the network moves from a top layer at which modules are interconnected to a bottom layer at which each SNP interacts with every possible other SNP through a hierarchy of intermediate layers composed of interconnected submodules, interconnected sub-submodules, and so on. The top-layer network presents a coarse-grained structure because its nodes are the mean values of genetic effects of all SNPs within a single module, whereas the bottom-layer networks are fine-grained in their structure where nodes stand for individual SNPs. Each module has a submodule network, and each submodule has a sub-submodule network, and so on, which forms a tree-like network. This network can code all possible SNPs from a mapping or GWAS experiment, thus, regarded as being omnigenic, through the implementation of functional clustering. Within each network, regardless of coarse-grained or fine-grained, variable selection is used to choose a small set of the most significant nodes that link with a given node, thus ensuring the sparsity of interactome networks.

## Worked Example

**Figure 2:**
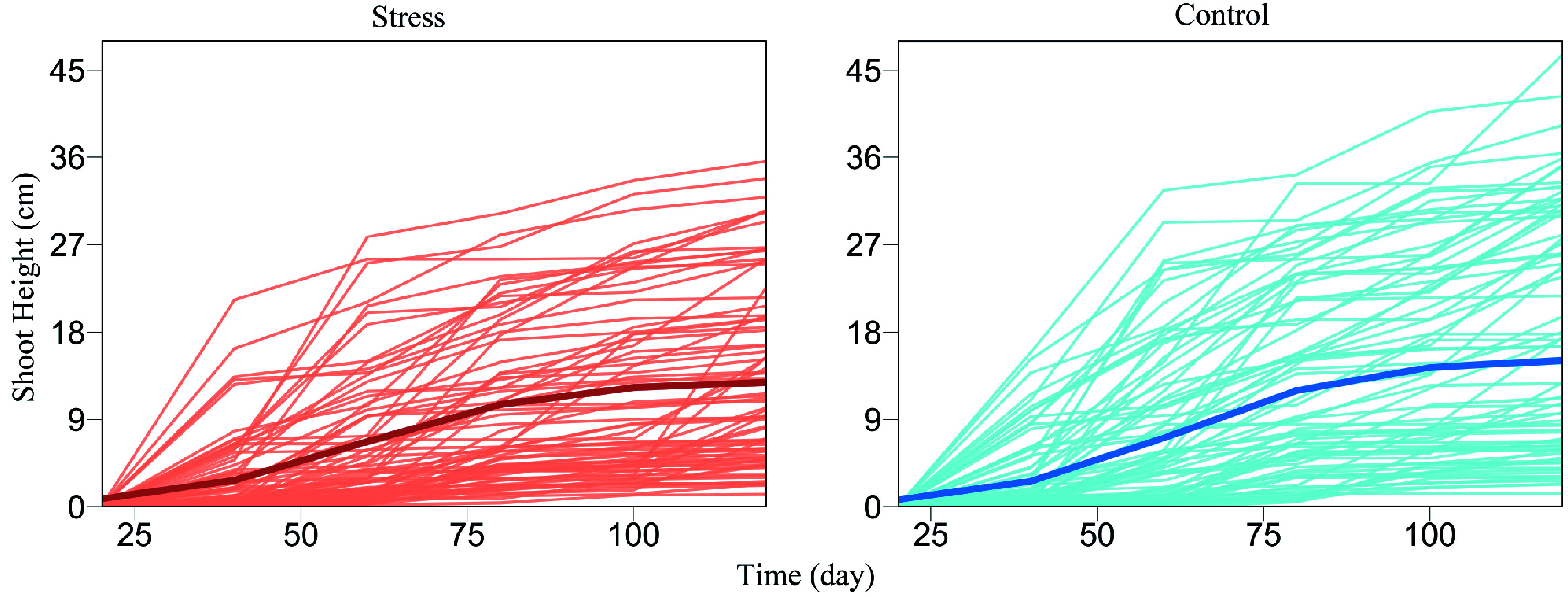
Growth curves of juvenile shoot heights (thin lines) for a GWAS population of Euphrates poplar cultured in salt-exposed (stress) and salt-free (control) conditions. The mean curve of young trees (thick line) under each condition is fitted by a logistic growth equation.

We demonstrate the utility and usefulness of FunGraph by analyzing a real data set from GWAS of Euphrates polar, a desert-adapted tree species^[47,48]^. Wang et al.^[31]^ gave a detailed procedure of how to generate this GWAS population and conduct an ecological genetic experiment using it. About 100 tree genotypes, genotyped by 272,719 quality SNPs, from this GWAS panel were cultured in salt-exposed (stress) and salt-free (control) conditions, where shoot heights were measured at a series of time points during early ontogeny. We plot shoot height growth trajectories for each tree genotype grown in stress and control conditions, from which it can be seen that height growth is well fitted by a logistic growth equation, despite a marked discrepancy in growth amount and form (Fig. 2). The difference of shoot height growth for a specific tree genotype under stress and control conditions, i.e., phenotypic plasticity, can be used as a proxy to assess the salt resistance of this genotype^[49,50]^. Our FunGraph is used to dissect the genetic architecture of salt resistance.

**Figure 3:**
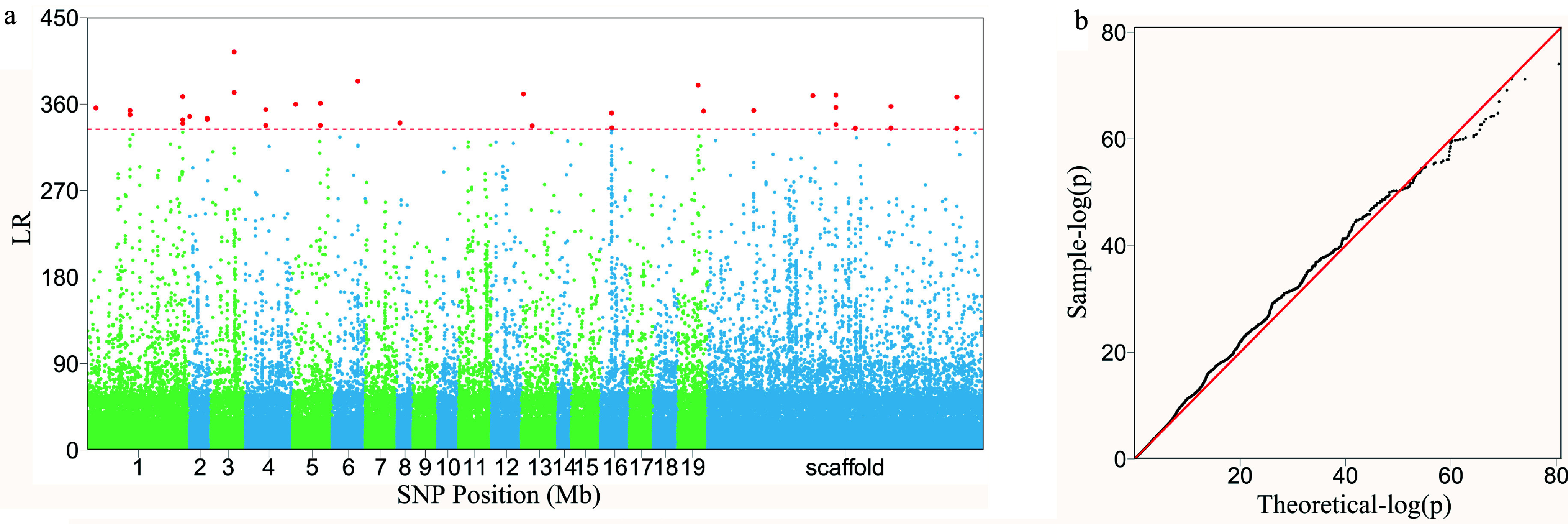
The detection of significant QTLs for salt resistance in Euphrates poplar by FunMap. (a) Manhattan plot of significance tests across 19 *Populus* chromosomes and scaffolds based on log-likelihood ratios (LR). The horizontal line is the critical threshold at 5% significance level determined by permutation tests. (b) QQ plot of statistical tests for the GWAS population.

**Figure 4:**
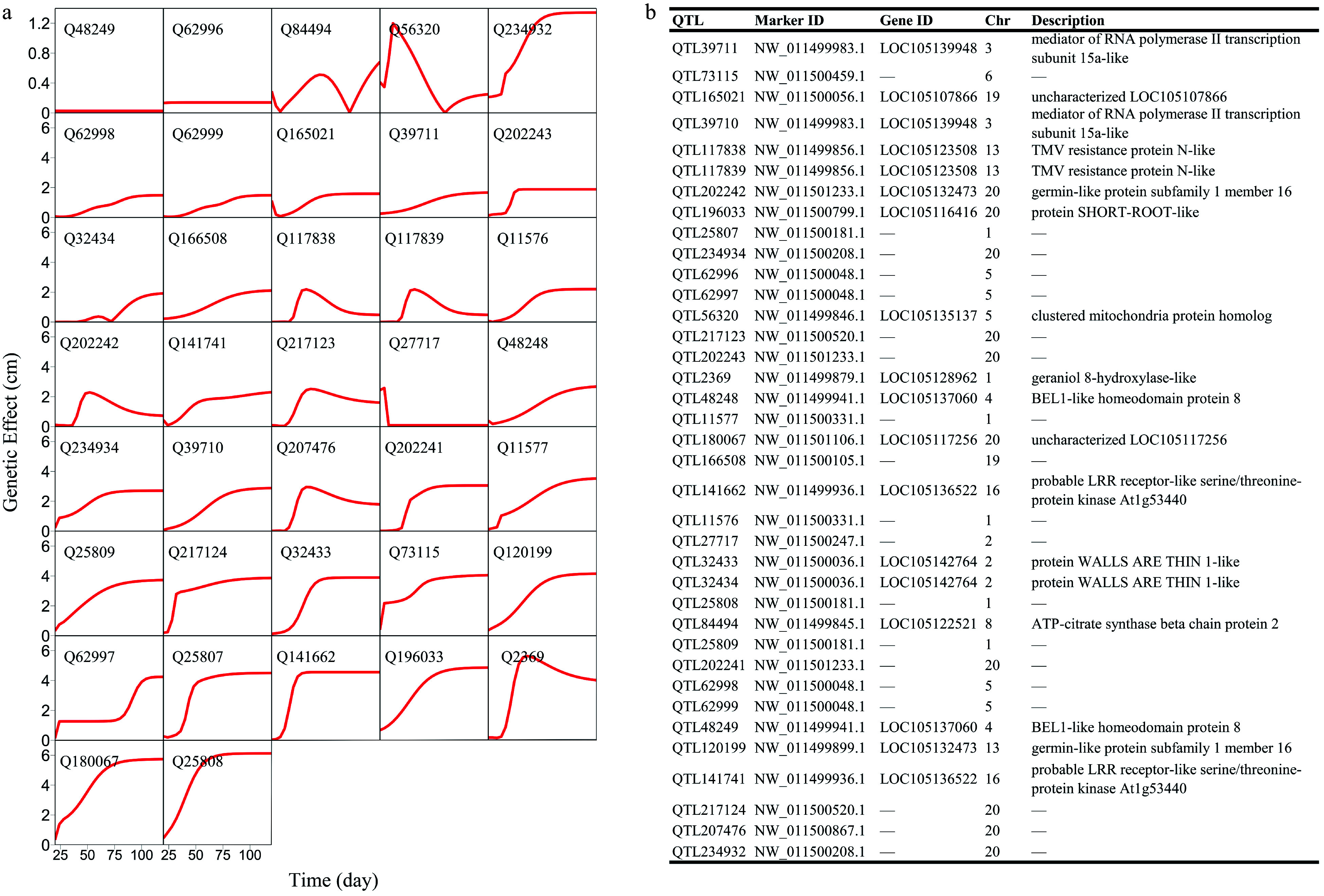
Genetic effect curves of (a) salt resistance QTLs and (b) their gene enrichment analysis.

Structural analysis of the GWAS panel shows no existence of subpopulations. FunMap identifies a small set^[36]^ of significant SNPs (i.e., QTLs) that mediate developmental phenotypic plasticity of shoot height growth, i.e., salt resistance (Fig. 3a). The Q-Q plot of significance tests suggests that FunMap is adequately accurate for QTL detection from this panel, without statistical inflation or deflation (Fig. 3b). GO analysis suggests that many of these detected QTLs are nearby candidate genes with known biological functions (Fig. 3c). QTLs identified display different temporal patterns of genetic effect on salt resistance; for example, some QTLs increase dramatically their effects with time, whereas some decrease their effects with time (Fig. 4). These QTLs will be highlighted and considered for their translation into practical breeding schemes, whereas those insignificant SNPs do not deserve further consideration. The above steps of QTL detection represent a general procedure for genetic mapping or GWAS.

**Figure 5:**
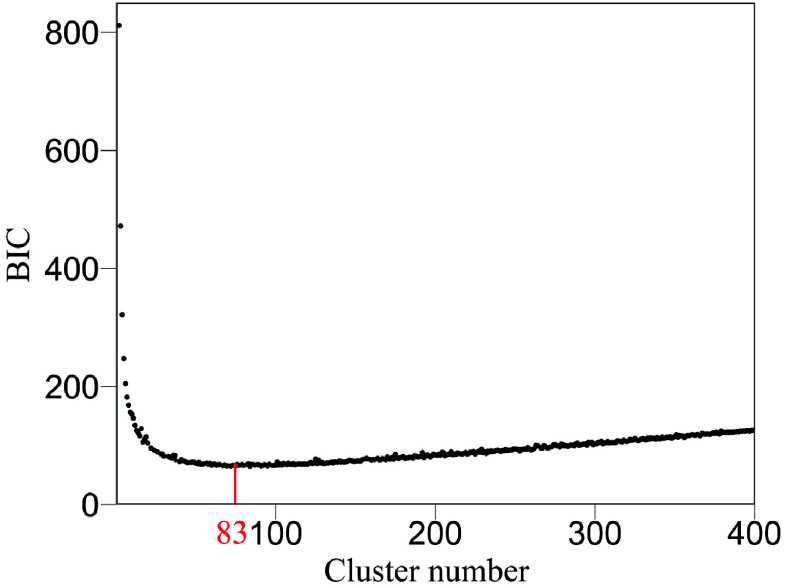
Classification of all SNPs into 83 modules (according to BIC) based on their similarity of temporal genetic effects.

**Figure 6:**
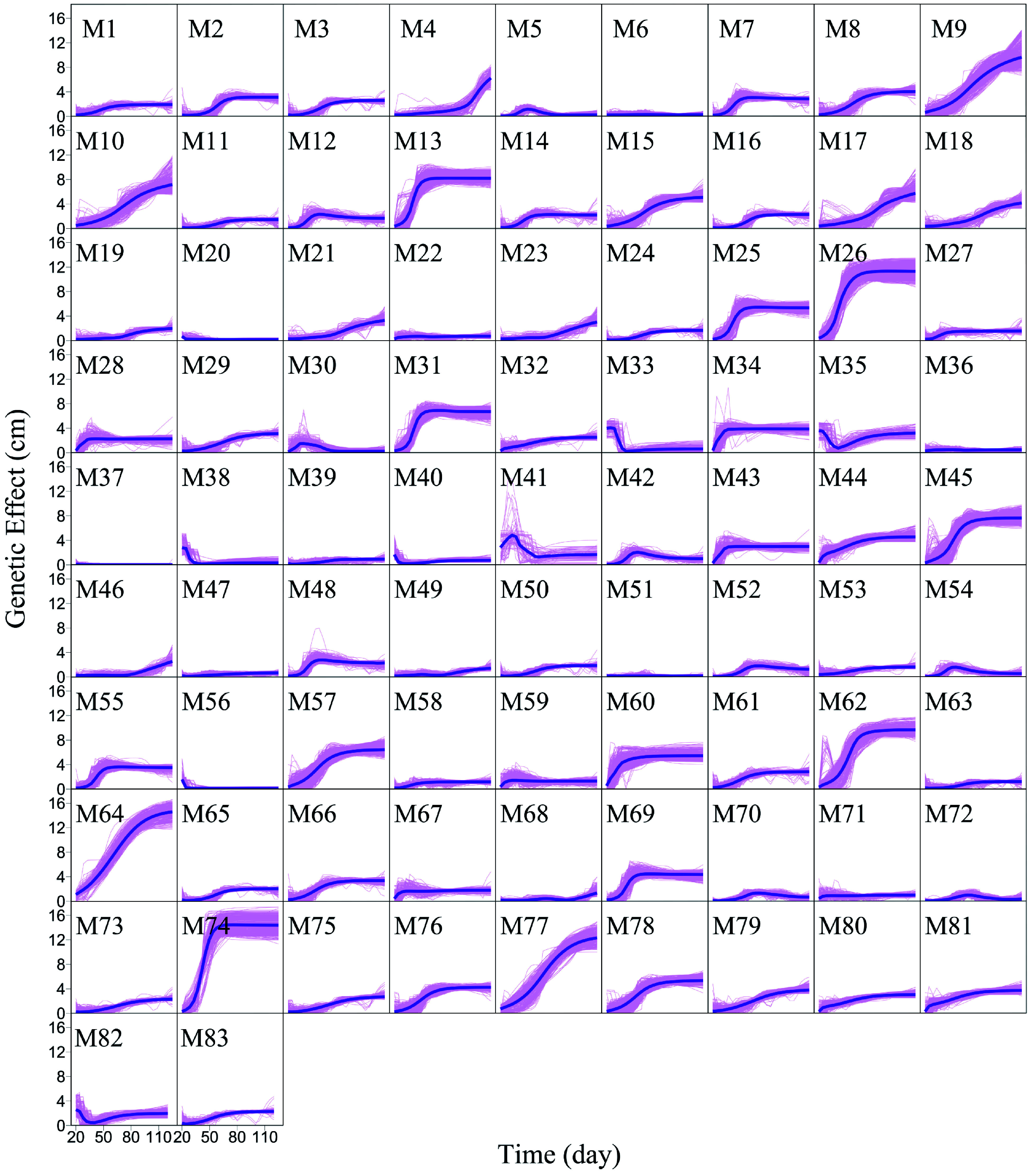
Mean genetic effect curves of each module M1 – M83. The background represents the genetic effect curves of all SNPs from a module.

We argue that QTLs detected from a reductionist-based approach may not adequately explain genetic variance in salt resistance^[29−32]^. It is crucial to reconstruct an omnigenic interactome network that cover all SNPs and their existing interactions by FunGraph, because such a network can more systematically capture the genetic architecture by revealing the significance of nonsignificant SNPs. This GWAS has 272,719 SNPs which in principle form a 272,719-node network for salt resistance. We do not attempt to reconstruct such a big network but dissolve it into different network communities by functional clustering. We classify 272,719 SNPs into 83 distinct modules based on their similarity of genetic effects according to BIC (Fig. 5), with each module representing a unique temporal pattern of genetic effect (Fig. 6). We further classify each module into distinct submodules and each submodule into distinct sub-submodules. Finally, we reconstruct a multilayer, multiplex, and multiscale interactome network that modulates the salt resistance of Euphrates poplar.

**Figure 7:**
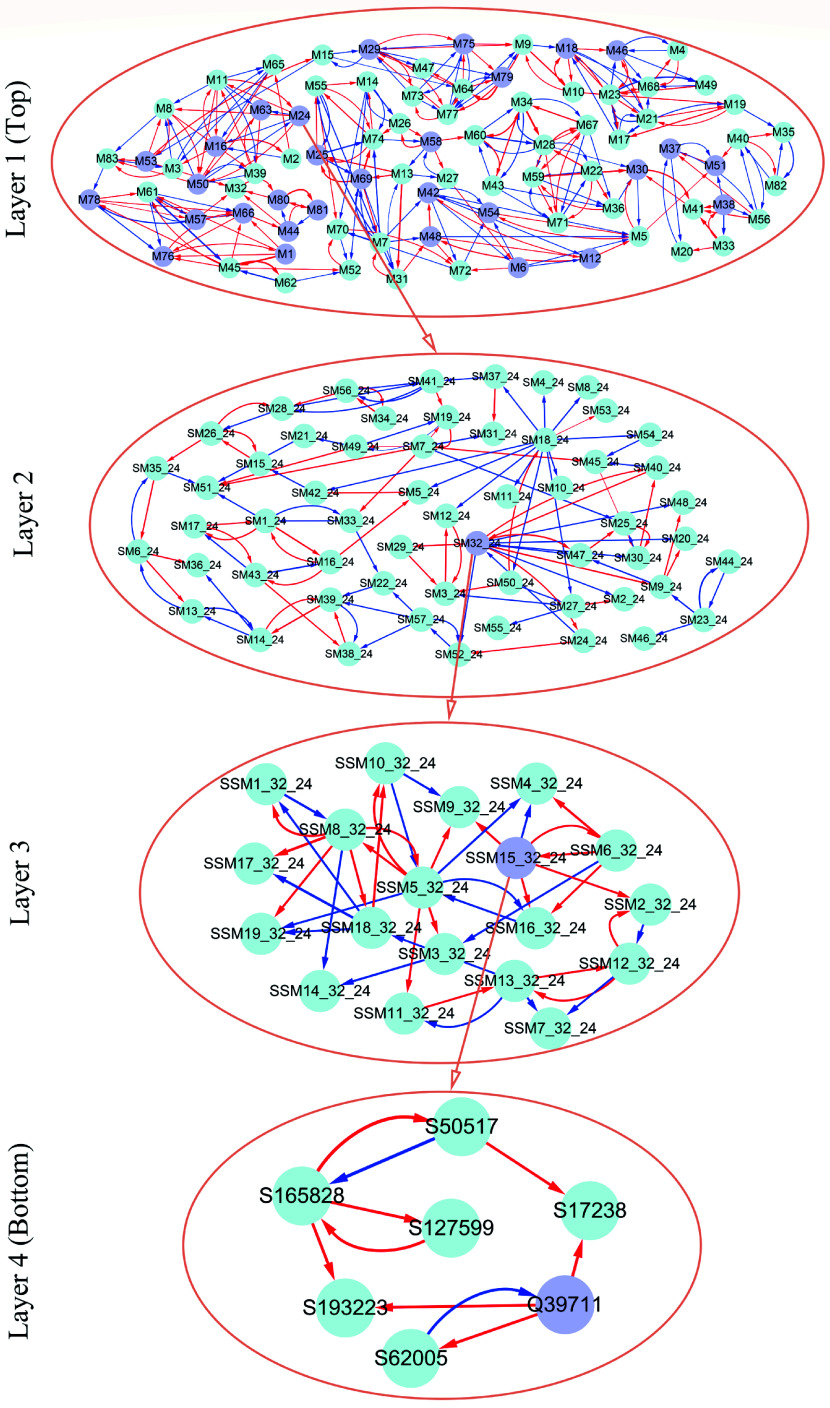
Multilayer interactome network for salt resistance in Euphrates poplar. At layer 1 (top) is the coarse-grained network among 83 modules. Layer 2 and 3 networks are the networks among submodules from module M24 and among sub-submodules from submodule SM32 of M24. At layer 4 (bottom) is the fine-grained SNP network from sub-submodule SSM15_32 of SM32. Arrowed red and blues lines stand for the positive and negative regulation of one SNP for the other SNP, respectively.

**Figure 8:**
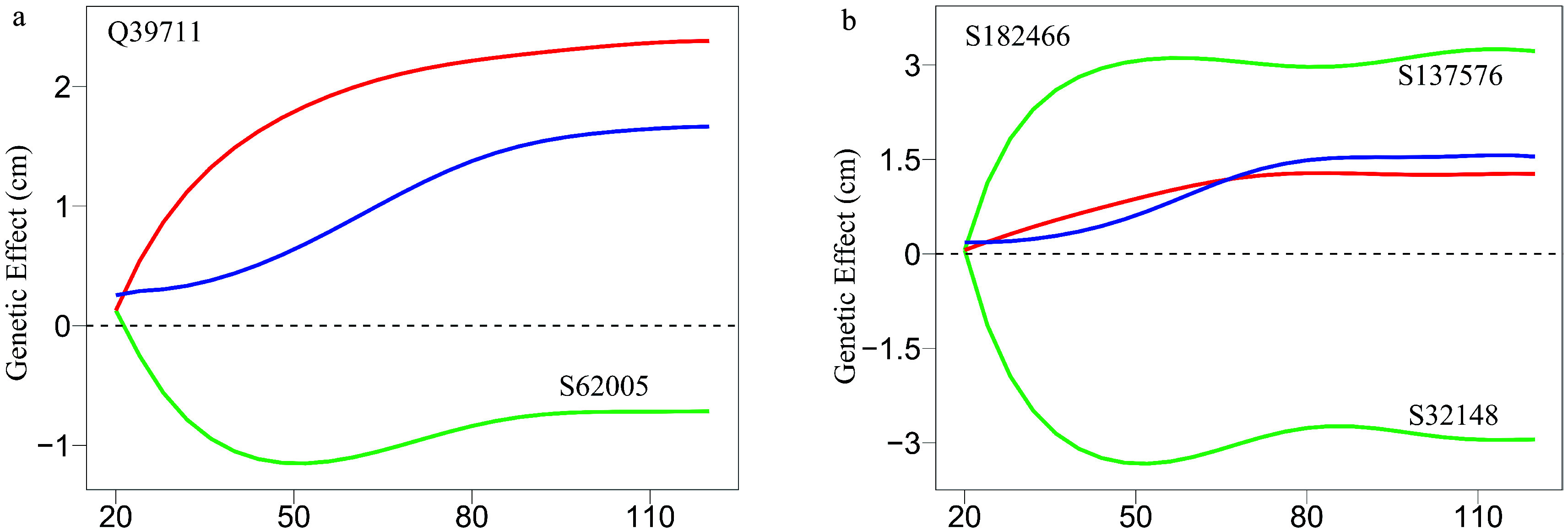
Decomposition of new genetic effects (blue line) into independent effects (red line) and dependent effects (green line) for (a) a QTL Q39711 and (b) an insignificant SNP S182466.

As an example, Fig. 7 illustrates part of such a multilayer interactome network. At the top layer is the 83-node coarse-grained interaction network among modules, with each module corresponding to a network community. Network communities are interconnected through the interactions between modules. Module M24 contains QTLs, which is split into 57 submodules, i.e., network sub-communities. We classify submodule SM32 that contains QTLs into 19 sub-submodules. Sub-submodule SSM15 contains QTL Q39711, for which we reconstruct a fine-grained SNP-SNP network. The genetic effect curve of Q39711 is dissected by evolutionary game theory. We find that this QTL has a strong independent genetic effect, which is compromised by negative regulation of SNP S62005 (Fig. 8a). In practice, by inhibiting the expression of this SNP as a negative regulator, Q39711 can fully be expressed to release its maximum genetic effect on salt resistance. We pick up an insignificant SNP S182466 from the same sub-submodule to characterize its independent and dependent effect curves (Fig. 8b). The net and independent effects of this SNP are subtle, which seems not to be useful for translation genetics. However, it simultaneously receives a similarly large positive and negative regulation from S137576 and S32148, which cancel each other out. Thus, by inhibiting negative regulator S32148, the role of positive regulator S137576 can be released so that insignificant S182466 can still be used in practice. The example of S182466 possibly explains the cause of missing heritability, a common phenomenon in GWAS. In summary, analyzing this Euphrates poplar GWAS data includes three key steps: functional mapping, functional clustering, and reconstructing multilayer networks. The entire process takes more than 40 days on a personal Dell commercial computer.

## Discussion

We argue that a network of cryptic epistasis is a possible cause for the unobserved genetic control mechanisms for stemwood growth. Increasing compelling evidence shows that epistasis contributes to the genetic architecture of complex traits^[21−25]^. Although tremendous effort has been made to detect epistasis, the efficiency of this pursuit may be very limited because an extremely large sample size is required^[28]^. We develop a functional network mapping model FunGraph to extract and excavate the hidden genetic architecture of complex traits by dissecting the detailed role of epistasis, but not largely relying on a hard-to-obtain sample size.

The statistical and biological power of FunGraph stems from the seamless integration of functional mapping, evolutionary game theory, and prey-predator theory. FunMap has been proven to be powerful for estimating time-varying net genetic variances explained by individual genes. Evolutionary game theory was introduced to formulate a regression model that expresses the net effect of a gene (response) as a function of its independent effect and dependent effects determined by a number of other genes (predictors) across time points (not across samples as usual). This formulation is constructed by a prey-predator model. We consider genetic architecture as a system composed of genome-wide genes whose number is exceedingly larger than the number of time points. We implement LASSO-based variable selection to resolve this 'curse of dimension', allowing us to select a small subset of the most significant predictors. The net genetic variance obeys a temporal pattern of changes from which infinite snapshots can be inferred, allowing variable selection to be implemented for any high dimensional pool of markers. This resolves a fundamental issue of an extremely large sample size required for epistatic detection.

FunGraph dissects the net effect of a gene into the independent effect that is expected to occur when this gene functions in a socially isolated condition and the accumulative dependent effect that result from the interactions of other genes with this gene. As such, a significant QTL is significant through three possible mechanisms. First, it displays a sizable independent effect on its own merit, not or only slightly affected by other genes. Second, this QTL has a little independent effect, but it contains a large overall dependent effect exerted by other genes. Third, both independent and dependent effects are large or even small, but they have the same sign to produce a pronounced accumulative effect. By analyzing the mapping data of Japanese larch, functional network mapping identified all these three mechanisms that guide the way significant QTLs affect stem height growth and diameter growth. Such a detailed characterization of how a QTL acts can help geneticists transform it into a tree improvement program, in which the genetic effect of this QTL can be strengthened or weakened by knocking out the expression of other genes that regulate it.

FunGraph can also address the question of why an insignificant marker is not significant. Indeed, an insignificant marker may not be necessarily insignificant if the independent and dependent effects do not cancel each other out. In other words, an insignificant gene can become significant if we can prevent cancellation between different types of effects. In addition, even if the insignificance of an insignificant gene is due to the negligible values of both independent and dependent effects, it can still play an important role in affecting complex traits through indirect paths if it serves as a strong regulator (leader) that regulates other genes. FunGraph has been able to chart direct and indirect roadmaps of each gene to affect the salt resistance of shoot height for Euphrates poplar. From these roadmaps, we can arouse the role of insignificant genes through activating or suppressing the expression of genes that interact with them.

Traditional mapping or association studies can estimate the proportion of the phenotypic variance explained by individual markers, i.e., marker-based heritability, but this heritability is due to the net genetic effect of a marker between its independent effect and dependent effect through epistasis with other markers. The merit of FunGraph lies in its power to estimate independent and dependent genetic variances separately, all of which are hidden in the heritability, called hidden heritability. Unlike tremendous efforts to detect missing heritability, i.e., an unexplained part of genetic variance^[51]^ by including rare variants and epigenetic marks among others^[52]^, hidden heritability can be retrieved by altering the pattern of gene-gene interactions. Because of its capacity to extract previously neglected genetic mechanisms underlying complex traits, FunGraph may upgrade and shift quantitative genetic theory from reductionist thinking to holistic, systems-oriented paradigm. First, it can refine the classic definition of epistasis as noncausal gene-gene interactions to a point at which epistasis is defined as a bidirectional, signed, and weighted measure. Qualitative classification and quantitative measure of epistasis can potentially stimulate geneticists to explore the biological, developmental, and molecular basis of gene-gene interactions. Second, traditional statistical genetics treats complex traits as a snapshot of biological processes to map and characterize genes that operate individually at a certain time and space. Different from this approach, systems genetics contextualizes biological processes as complex systems and divides them into a series of organizational functionality from genes to organismal properties^[53]^. At the heart of systems genetics are network models that can pack and organize various interacted components into graphs. FunGraph, accompanied by subsequent modifications and perfections in a variety of areas, such as the high-dimensionality of genome-wide genes, high-order gene interactions, gene-environment interactions, and phenotypic plasticity, could provide a novel tool to comprehend the mechanistic understanding of complex traits and quantitate the mechanistic process of how complex traits are formed, progressed, and altered across spatiotemporal scales.

## Data and code availability

All the data and code used in this paper are available at https://github.com/BeijingCCB/EuphratesFunGraph.
